# LIN28‐Targeting Chromenopyrazoles and Tetrahydroquinolines Induced Cellular Morphological Changes and Showed High Biosimilarity with BRD PROTACs

**DOI:** 10.1002/cmdc.202400547

**Published:** 2024-11-12

**Authors:** Mao Jiang, Nicole Giannino, Georg L. Goebel, Sonja Sievers, Peng Wu

**Affiliations:** ^1^ Chemical Genomics Centre Max Planck Institute of Molecular Physiology Otto-Hahn Str. 11 Dortmund 44227 Germany; ^2^ Department of Chemical Biology Max Planck Institute of Molecular Physiology Otto-Hahn Str. 11 Dortmund 44227 Germany; ^3^ Faculty of Chemistry and Chemical Biology TU Dortmund University Otto-Hahn Str. 6 Dortmund 44227 Germany; ^4^ Compound Management and Screening Center Max Planck Institute of Molecular Physiology Otto-Hahn Str. 15 Dortmund 44227 Germany

**Keywords:** LIN28 inhibitor, Chromenopyrazole, Tetrahydroquinoline, Cell painting assay, PROTAC degrader

## Abstract

The probing of small molecules with heterocyclic scaffolds covering unexplored chemical space and the evaluation of their biological relevance are essential parts of forward chemical genetics approaches and for the development of potential small‐molecule therapeutics. In this study, we profiled sets of chromenopyrazoles (CMPs) and tetrahydroquinolines (THQs), originally developed to target the protein–RNA interaction of LIN28–*let‐7*, in a cell painting assay (CPA) measuring cellular morphological changes. Selected LIN28‐inactive CMPs and THQs induced cellular morphological changes to different extents. The most CPA‐active CMPs **2** and **3** exhibited high bio‐similarity with the LCH and BET clusters, while the most CPA‐active THQs **13** and **20** indicated a mechanism of action beyond the currently established biosimilarity clusters. Overall, this work demonstrated that CPA is useful in revealing “hidden” biological targets and mechanisms of action for biologically inactive small molecules, which are CMPs and THQs targeting the RNA‐binding protein LIN28 in this case, evaluated in target‐based strategies. When compared with annotated reference compounds, CMP **3** exhibited a high biosimilarity with the dual BRD7/9 degrading PROTAC VZ185, suggesting that CPA could potentially function as a new phenotypic approach to identify degrader molecules.

## Graphical abstract

Phenotypic profiling of chromenopyraoles (CMPs) and tetrahydroquinolines (THQs) that were developed to target the protein‐RNA interaction of LIN28–let‐7 in cell painting assay revealed that selected LIN28‐inactive CMPs and THQs induced significant cellular morphological changes and showed high bio‐similarity with the LCH and BET clusters and BRD7/9‐degrading PROTACs.@mpimoph@tu_dortmund@PengWu_Lab

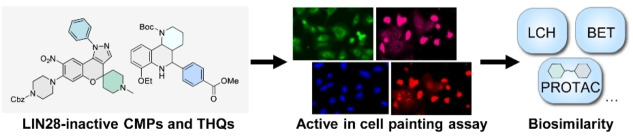

## Introduction

**Figure 1:**
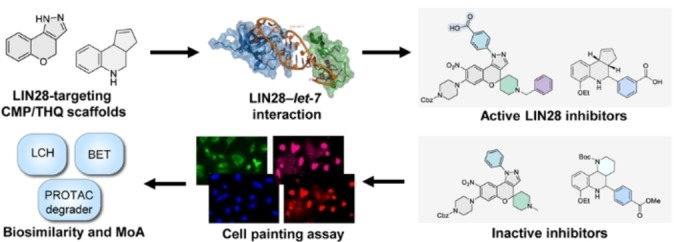
The workflow of the study evaluating the biological targets and mechanism of action for the inactive LIN28 inhibitors with either a chromenopyrazole (CMP)‐ or a tetrahydroquinoline (THQ)‐containing scaffold via the cell painting assay measuring morphological changes.

Small molecules with a fused chromenopyrazole (CMP) and tetrahydroquinoline (THQ) scaffold have exhibited a wide range of biological and pharmacological activities. For example, CMP and associated conjugates have been reported as inhibitors of the oncogenic microRNA‐binding protein LIN28,[^1^, ^2^] selective agonists and fluorescent ligands against Cannabinoid type 2 receptor (CB_2_R) associated with pain and inflammatory disorders,[^3^, ^4^] and inhibitors against Cryptosporidium IMP dehydrogenase for treating Cryptosporidium parasites.^[5]^ Recently, we reported a series of spirocyclic CMPs as small‐molecule inhibitors disrupting the protein–RNA interaction of LIN28 and *let‐7* miRNA.^[6]^ The most active compound **1** (FB‐5), which features a spiropiperidine scaffold, led to increased mature *let‐7* levels in choriocarcinoma cells expressing LIN28 and induced a decrease of expression of *let‐7* target oncogenes.^[6]^ A series of structurally modified analogues were subsequently synthesized based on the chromenopyrazole scaffold of compound **1** with the aim of obtaining the minimum core scaffold that would be essential for LIN28 inhibition (Figure 1A). Although we did not succeed in obtaining such a minimum core scaffold as all the structurally modified analogues showed either greatly decreased or a complete loss of LIN28 inhibitory activity, it is intriguing to observe that the thus obtained chromenopyrazoles retained antiproliferation activities against cancer cells, such as compound **2** without the Bn group, compound **3** without the carboxylic acid group, and compound **4** without the spirocyclic piperdine moiety, which triggered us to evaluate the unknown hidden targets and mechanism for the LIN28‐inactive CMPs. Likewise, the THQ is another scaffold that has been reported in small‐molecule inhibitors disrupting the interaction between LIN28 and *let‐7* microRNAs.[^7^, ^8^] Given the fact that the antiproliferation activity against cancer cells for the LIN28‐inhibiting THQs did not correlate well with the LIN28‐inhibitory potency, we propose that unidentified anticancer targets contributed to the observed anticancer activity of the LIN28‐inactive THQs.^[7]^

Triggered by the unexplained discrepancy between the observed LIN28 inhibitory activities and the anticancer activities for the CMPs and THQs, we, therefore, resorted to the phenotypic screening approach named cell painting assay (CPA) to probe the potential biological targets and anticancer mechanism for the involved CMPs and THQs. CPA is an image‐based morphological profiling assay measuring cellular morphological changes via multiple fuorescent dyes.^[9]^ Different from many image‐based assays that extract a limited number of cellular features, CPA quantifies a very large collection of more than 1500 morphological features in multiple channels in a relatively unbiased manner.^[10]^ Upon treated with small molecules, the induced cellular morphological changes were visualized in combination with high‐content imaging and automated analysis to generate morphological fingerprints for each compound, therefore, CPA has been successfully applied as an emerging phenotypic approach to study the mechanisms of action for bioactive small molecules.[^11^, ^12^, ^13^] Such an unbiased profiling approach enabled the detection of bioactivities of small molecules in a broader range and offered the possibility to directly identify the putative biological targets and study the mechanisms of action by comparing with predetermined and annotated bio‐clusters and reference compounds.,[^14^, ^15^, ^16^] which represents an alternative morphological‐based method distinct from the widely applied target‐based and phenotypic‐based approaches to study bioactive small molecules. Recently, CPA was applied to show the phenotypic changes of proteolysis targeting chimeras (PROTACs)^[17]^ and uncover the association between the RNA‐binding protein YTHDF2 and aurora kinase signalling induced by dual YTHDF2‐ and aurora kinase‐targeting PROTACs.^[18]^

In this work, we applied the CPA to perform the morphological profiling of LIN28‐targeting CMPs and THQs and studied the potential mechanism of action and biological targets for the LIN28‐inactive molecules, which revealed that the LIN28‐inactive but CPA‐active CMPs showed high biosimilarity with the established clusters, while the CPA‐active THQs may have a mechanism of action (MoA) that is beyond the established clusters. The cluster biosimilarity for the CMPs echoed the results from a subsequent comparison analysis with annotated reference compounds, which led to an intriguing finding of a high biosimilarity observed between a CMP and a PROTAC with potent and selective degradation activity for the bromodomain‐containing proteins BRD7 and BRD9 (Figure 1).

## Results and Discussion

### LIN28‐targeting Scaffold Minimization and Synthesis

**Figure 2:**
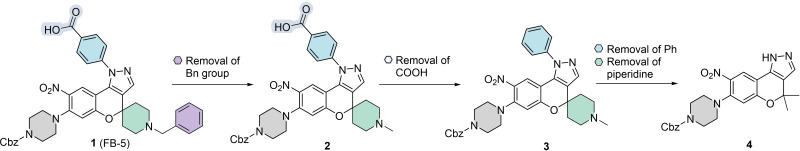
Bioactive CMPs that were probed in the core‐scaffold search efforts by removing structural moiety based on the previously reported LIN28 inhibitor **1**, leading to compounds **2**–**4** with greatly decreased or complete loss of activity against the LIN28–*let‐7* inhibition.

To obtain the minimum core scaffold based on the CMP in our LIN28‐targeting strategy, it was rationalized based on the established structure‐activity relationship that the carboxylic acid group on the *N*‐phenyl moiety and the presence of the aromatic groups to form stacking interactions are essential for LIN28 inhibition.^[19]^ Therefore, it is expected that the removal of the Bn group (CMP **2**), the carboxylic acid group (CMP **3**), and the phenyl group at the *N*‐1 position and the spirocyclic piperidine moiety (CMP **4**) all led to chromenopyrazole analogues with a decreased or a complete loss of LIN28 inhibitory activity (Figure 2).

**Scheme 1:**
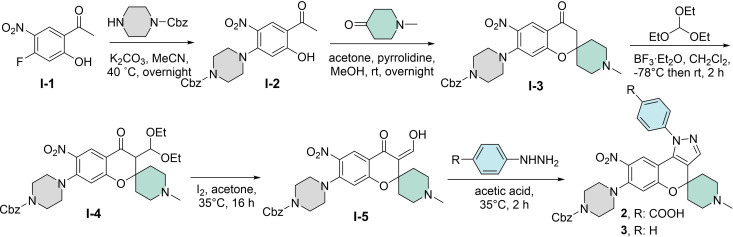
Synthesis of the spirocyclic CMP‐containing compounds **2** and **3**.

The small‐molecule CMP and THQ inhibitors that were evaluated in this study were obtained based on synthetic routes from our previous reports.[^2^, ^6^, ^7^] For example, the spirocyclic chromenopyrazoles **2** and **3** were synthesized based on the route that we previously reported,^[6]^ by using 1‐methylpiperidin‐4‐one as the aldehyde component to react with the Cbz‐piperazine‐installed acetophenone **I‐2** to yield compound **I‐3** with the spiro[chromane‐2,4′‐piperidin]‐4‐one scaffold. The final condensation step with the phenylhydrazine or hydrazine led **2** and **3** with the chromenopyrazole core scaffold (Scheme 1). The chromenopyrazole **4** with the *gem*‐dimethyl moiety, instead of the spirocyclic moiety, at the 4‐position of the core scaffold was synthesized based on another route that we previously reported.^[2]^

The CPA was performed using the human osteosarcoma U2OS cells by exposing to different concentrations of the small‐molecule CMPs or THQs (ranging between 1 μM and 50 μM), incubated for 20 h before the staining of eight cellular compartments (being nucleus, nucleoli, mitochondria, endoplasmic reticulum, Golgi, plasma membrane, actin cytoskeleton, and cytosolic RNA) using six different dyes. A total of 579 morphological features were extracted employing high‐content screening and image analysis to generate morphological fingerprints,^[20]^ which were used to identify biologically relevant similarities and differences among samples to determine the induction value which describes the fraction of significantly altered parameters in comparison to the vehicle control (in percent) and is used as a quantitative measure of bioactivity. Generally, compounds that showed an induction value of more than 5 % were considered biologically active.^[21]^

### CPA Active CMPs and THQs and Inactive LIN28 Inhibitors

**Figure 3:**
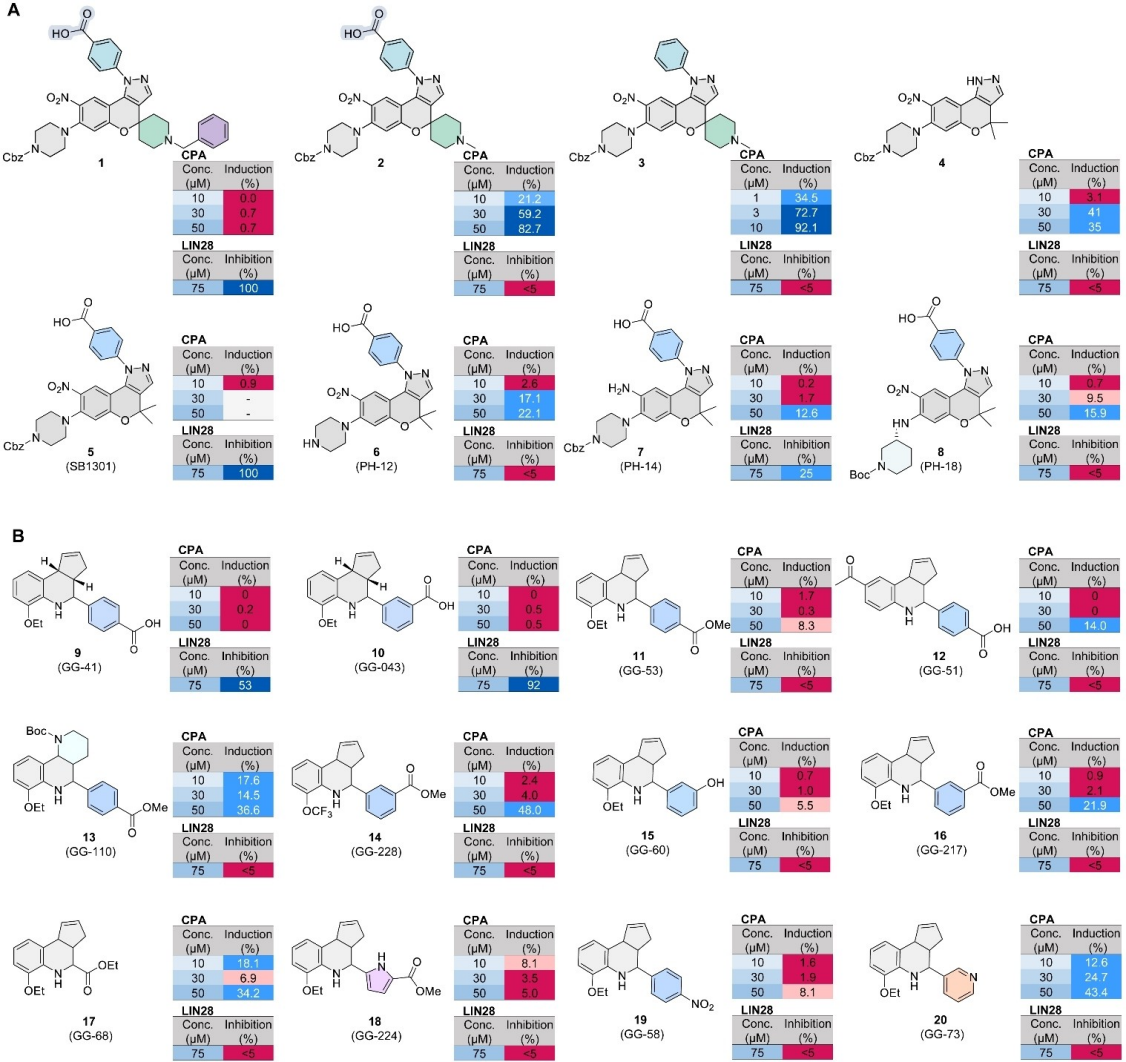
Results of testing the CMPs and THQs in the cell painting assay (CPA). (A) Induction value (%) in CPA for the CMPs tested in different concentrations, together with the inhibitory activity against the LIN28–*let‐7* interaction tested at 75 μM in the fluorescence‐polarization assay (FP). Compounds that showed an induction value of more than 5 % were considered biologically active. (B) Induction value (%) in CPA for the THQs tested in different concentrations, together with the inhibitory activity against the LIN28–*let‐7* interaction tested at 75 μM in the FP. For induction values of>50 % are shown in blue, for induction values between 10 and 50 % are shown in light blue, and for induction values between 5 and 10 % are shown in light magenta, and values <5 % are shown in magenta.

The CPA result of the CMPs **1–4** and a few structurally related analogues **5**–**8** that were previously evaluated as LIN28 inhibitors (Figure S1) showed a clear discrepancy between their LIN28 inhibitory potency and their ability to induce cellular morphological change evaluated judged by the induction value. The CPA results, together with the LIN28 inhibitory activities of the involved compounds, are summarized in Figure 3A. Among the eight CMPs that we tested in the CPA, the spirocyclic CMP compound **1** and the reported inhibitor **5** (SB1301) were the only active LIN28 inhibitors that showed complete inhibition against the LIN28–*let‐7* interaction at 75 μM, however, they did not induce cellular morphological changes in the CPA. The LIN28‐inactive compounds **2** and **3** showed the most potent activity in CPA, especially for compound **3**, which showed an induction of 34.5 % even at a low concentration of 1 μM, the only compound that showed >5 % induction among the tested compounds in this study. This is a clear indication that the observed CPA activity was not associated with LIN28 inhibitory activity as compound **3** has no carboxylic acid, which is reported to be essential for LIN28 inhibition, in its CMP structure.^[2]^ In addition to compounds **2** and **3**, the other CMPs **4**,**6**,**7**,**8** showed different extents of induction activity ranging between 12.6 % and 35 % at the highest tested concentration of 50 μM.

**Figure 4:**
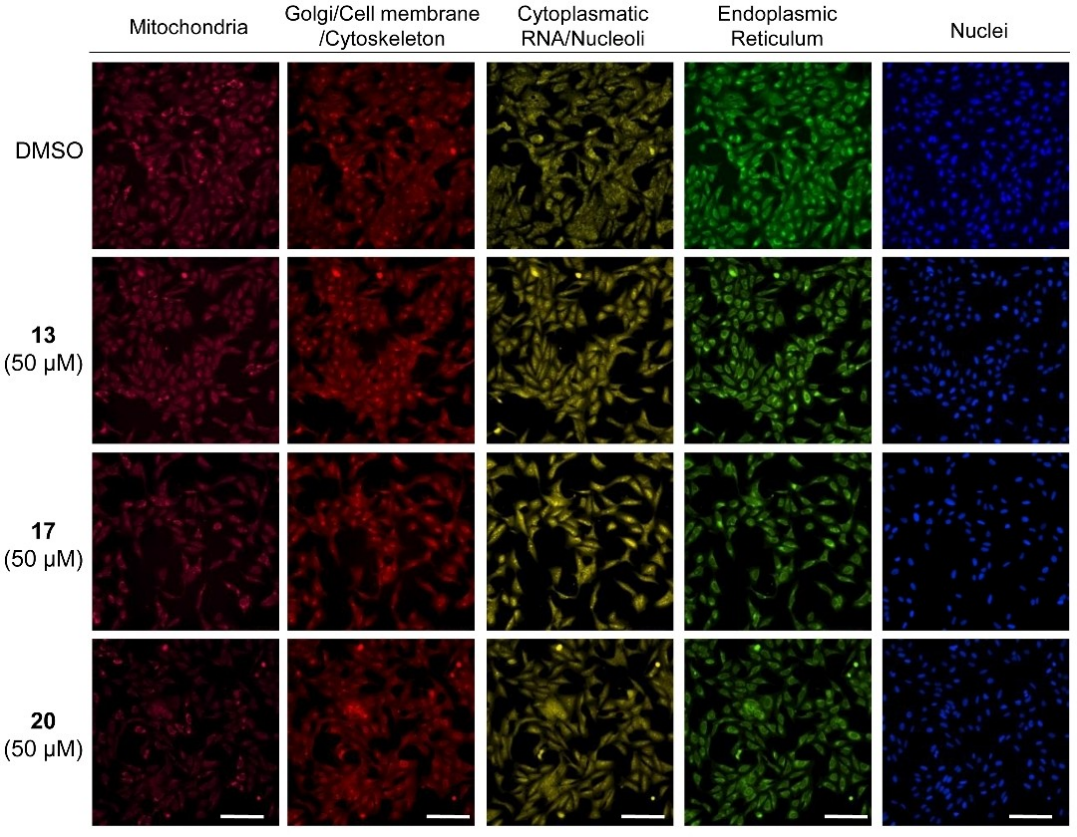
Selected images from the CPA analysis showing the cellular morphological change of U2OS cells upon treatment with the THQs **13**, **17**, and **20** at 50 μM. DMSO was used as the control. Scale bar, 150 μm for all images.

The CPA result for the THQs **9**–**20** echoed the discrepancy between the LIN28 inhibitory activity and induction values as observed for the CMPs (Figure 3B, Figure 4). Compounds **9** and **10** with either a 3‐ or 4‐carboxylphenyl substituent were the only two active LIN28 inhibitors but both were inactive in the CPA. Among the 12 tested THQs, only compounds **13** with a methoxy carboxylphenyl group and compound **20** with a pyridine‐3‐yl group were active in CPA at the lowest tested concentration of 10 μM, with an induction being 17.6 % and 12.6 %, respectively. In addition to compounds **13** and **20**, compounds **14**, **16**, and **17**, all of which harbor either a methoxycarboxyl or an ethoxycarboxyl group instead of a carboxylic acid group, were active in the CPA at the highest tested concentration of 50 μM. The lack of the carboxylic acid group in the CPA‐active THQs and the overall structure‐activity relationship for the CPA activity is another reflection that the observed CPA activity is not associated with LIN28 targeting. Despite the discrepancy between CPA activity and LIN28 inhibition, all tested compounds showed no cytotoxicity at the tested concentrations (>50 % of the relative cell counts in comparison with that of the cells without compound treatment, data not shown here), which is not surprising to observe that the THQs showed minimum inhibition in an antiproliferation assay against cancer cells, even for the active LIN28 inhibitors **9** and **10**.^[7]^

### CMPs Shared a High Biosimilarity with LCH and BET Clusters

Given the non‐overlapping results of CPA activity and LIN28 inhibition, we wondered what potential biological targets and mechanisms of action could be involved for the observed CPA‐active but LIN28‐inactive compounds. Therefore, we proceeded with the biosimilarity analysis based on the CPA data. The generated fingerprints from CPA were compared to obtain a biosimilarity score.^[22]^ Compounds that display similar fingerprints are expected to have a high level of biosimilariy, i. e. to address the same targets or share the same mode of action (MoA).^[23]^ The score was calculated based on Person's correlation distance. A high similarity in their fingerprints indicated a potential shared mechanism of action (MoA) or biological targets. Considering the induction values as shown in Figure 3, we focused on the CMPs **2** and **3** and THQs **13** and **20** for the following investigations (Figure S2).

**Figure 5:**
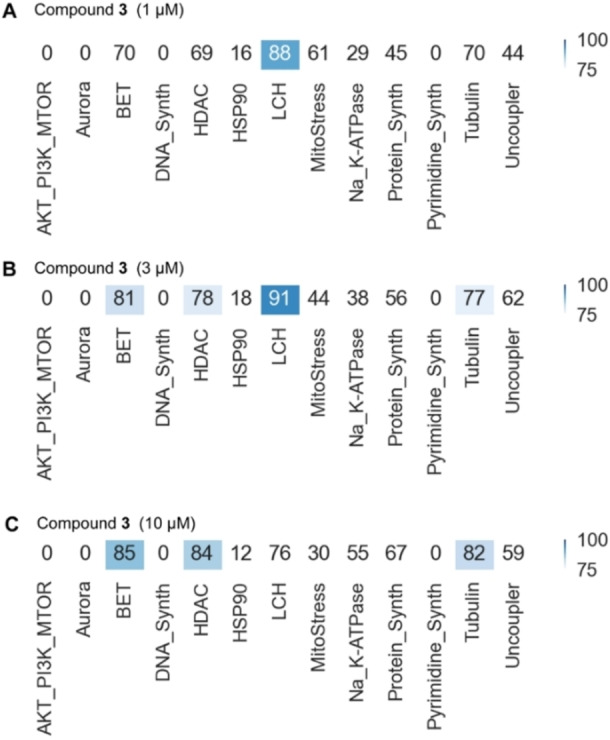
Subprofile analysis of the CMP **3**’s cluster biosimilarity at different concentrations. Compound **3** showed a high biosimilarity with the LCH cluster at 1 and 3 μM, and a high biosimilarity with the BET cluster was observed at 10 μM. The 13 established clusters are associated with AKT/PI3 K/MTOR, aurora kinase, bromodomain and extra‐terminal domain (BET), DNA synthesis, histone deacetylase (HDAC), heat shock protein 90 (HSP90), lysosomotropism/cholesterol homeostasis (LCH) regulation, mitochondrial stress regulation, Na^+^/K^+^ ATPases, protein synthesis, de novo pyrimidine biosynthesis, tubulin, and uncoupling of the mitochondrial proton gradient.

Firstly, we performed the biosimilarity analysis between the CPA active compounds with annotated clusters, which were defined by assessing morphological changes induced by characterized compounds displaying similar fingerprints from the CPA analysis.^[14]^ More specifically, the annotated clusters were defined keeping only the morphological features that are shared among a selected collection of compounds to generate a subprofile, for which a high similarity of>85 % to a defined subprofile generally enabled the biological annotation of uncharacterized compounds with unclear MoA. Recently established clusters include mitochondrial stress cluster and aurora kinase inhibition cluster.[^24^, ^25^] Here, we performed the subprofile analysis for the four compounds, each at different concentrations, by comparison with 13 established bio‐clusters as shown in Figure 5 and Figures S3–S5.

The most active compound CMP **3** showed the highest biosimilarity with the cluster of lysosomotropism/cholesterol homeostasis regulation (LCH) at both 1 μM and 3 μM, while the high biosimilarity with the clusters of bromodomain and extra‐terminal domain (BET) and histonedeacetylase (HDAC) was observed at 10 μM. The bioactivity cluster analysis for compound **2** showed a consistent result with that of CMP **3**, i. e., the highest similarity with the LCH and BET clusters at 30 μM and 50 μM.

Overall, the results indicated a high biosimilarity of the CMPs with the LCH cluster at different concentrations, which indicated that the LIN28‐inactive CMPs’ antiproliferation activity against cancer cells can be attributed to their lysosomotropic property associated with disturbed cholesterol homeostasis in cancer cells.[^26^, ^27^, ^28^]

### MoA of THQs Beyond the Established Clusters

In contrast to the CMPs **2** and **3**, the THQs **13** and **20** did not show a high biosimilarity with any of the established clusters at the three tested concentrations ranging from 10 μM to 50 μM as all observed biosimilarity were below 85 %. The subsequent comparison with annotated reference compounds in our in‐house database did not yield any target information judged by the less than 85 % biosimilarity observed with reference compounds. Overall for the THQs, the lack of bisimilarity with the established clusters or an annotated reference compound indicated that the potential biological targets and MoAs contributing to the observed induction activity in CPA would need to be addressed alternatively, for example, by employing a proteomic analysis.^[29]^

### CMP 3 Shared a High Biosimilarity with a Dual BRD7/9 PROTAC Degrader

Stimulated by the observed high biosimilarity with the LCH and BET clusters for the CMP compound **3** even at low concentrations, we proceeded to scrutinize the biosimilarity with annotated reference compounds for compound **3** at different concentrations, which showed the polypharmacological properties of CMP **3** with annotated reference compounds of diverse activities. At 1 μM, compound **3** showed a high biosimilarity with a set of bioactive compounds of different MoA. The top five reference compounds that shared the highest biosimilarity are the selective 5‐HT1B and 5‐HT1D serotonin receptor antagonist GR127935 (biosimilarity 93.0 %), the weak inhibitor of phospholipase C U73343 (biosimilarity 92.4 %), the high affinity muscarinic ligand aminobenztropine (biosimilarity 92.1 %), the sterol 14‐demethylase inhibitor terconazole (biosimilarity 91.8 %), and the potent and selective inhibitor of the BRPF1 bromodomain GSK6853 (91.6 %). At 10 μM, compound **3** showed high biosimilarity with a different set of reference compounds with the top three being the multi‐target kinase inhibitor ponatinib (biosimilarity 87.6 %), the potent and selective BET inhibitor CF53 (biosimilarity 87.6 %), and the potent and selective Lck inhibitor RK‐24466 (biosimilarity 87.0 %).

**Figure 6:**
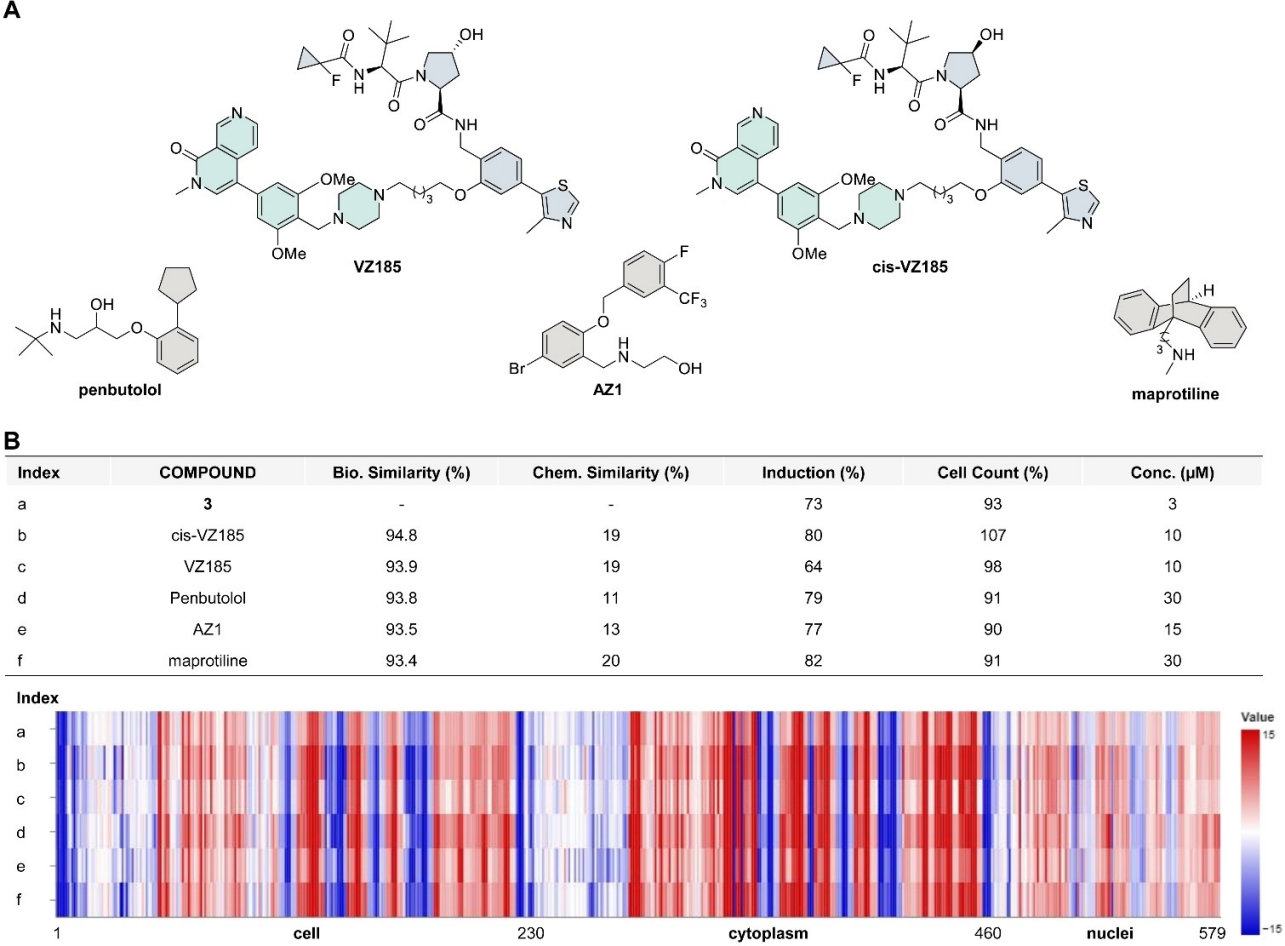
Biosimilarity of the CMP **3** with annotated reference compounds. (A) The five reference compounds that shared the highest biosimilarity with the CMP **3** at 3 μM. The five compounds include the dual BRD7 and BRD9 PROTAC degrader VZ185, the inactive degrader PROTAC cis‐VZ185, the adrenergic receptor antagonist penbutolol, the USP25/28 inhibitor AZ1, and the selective noradrenalin reuptake inhibitor and antidepressant maprotiline. (B) The biosimilarity chemical similarity, induction, relative cell counts of compound **3** and the five reference compounds. Together with the heatmaps for the **3** and the five reference compounds in the CPA. The set of 579 parameters is divided into parameters related to cell (1–229), cytoplasm (230–461) and nuclei (462–579). Values were normalized to that of the DMSO control. Blue: decreased parameters; red: increased parameters.

What we found of particular interest was the list of reference compounds that shared the highest biosimilarity with compound **3** at 3 μM (Figure 6). The top two compounds that showed the highest biosimilarity (94.8 % and 93.9 %) were the proteolysis‐targeting chimera (PROTACs) VZ185, which was reported a potent and selective dual BRD9 and BRD7 degrader (DC_50_: 1.8 nM and 4.5 nM, respectively), and its inactive analogue cis‐VZ185.^[30]^ In addition to the PROTAC VZ185 and the inactive analogue cis‐VZ185, compound **3** at 3 μM showed a high biosimilarity with the adrenergic receptor antagonist penbutolol (93.8 %), the USP25/28 inhibitor AZ1 (93.5 %),^[31]^ and the noradrenain reupdate inhibitor maprotiline (93.4 %). Overall, the high biosimilarity with the BRD degrader and BRD inhibitors corresponded to the observed subprofile biosimilarity with the BET cluster.

## Perspective and Conclusions

In this work, we evaluated small molecules with either a CMP or THQ scaffold, which were initially developed in‐house as LIN28 inhibitors, in the CPA measuring cellular morphological changes. The discrepancy between the LIN28 inhibitory potency and the CPA induction activity was clearly shown for both the CMPs and THQs. Specifically, the LIN28 inactive CMPs **2** and **3** showed high induction values at different concentrations ranging from 1 to 30 μM, the same for the LIN28‐inactive THQs **13** and **20**. Comparison of the CPA fingerprint with the established subprofile clusters revealed that CMPs **2** and **3** shared high biosimilarity with the LCH and BET clusters, while the lack of high biosimilarity with any of the established clusters for the CPA‐active THQs **13** and **20** indicated that an uncovered mechanism of action beyond the described clusters is involved. With the establishment of new subprofile clusters in the cell painting assays, such cases of lack of overlapping clusters can be gradually addressed.

An exciting finding from this study is the observed high biosimilarity between the CMP **3** and the PROTAC degrader VZ185 that showed potent and selective degradation activity for BRD9 and BRD7. One limitation of the current study lies in the lack of validation whether compound **3** can induce BRD9/7 degradation. Although compound **3** and the PROTAC VZ185 shared high biosimilarity, it has to be noted that the inactive PROTAC analogue cis‐VZ185 also demonstrated the equivalent level of high biosimilarity. Thus, it will be meaningful to demonstrate the feasibility of potentially identifying degrader molecules via CPA with additional experimental data.

Our recent study reported that CPA can be used to characterize dual targeting PROTACs of the RNA‐binding protein YTHDF2 and the aurora kinase.^[18]^ This study focuses on the reverse direction of retrieving compounds of unclear MoA but sharing high biosimilarity with characterized PROTACs. Taken together, these two studies complement the scope of applying CPA for the evaluation of both classical small molecules and new degrading chemical modalities represented by PROTACs.

## Experimental Section

**LIN28 protein purification**. Human LIN28 A 16–187 was subcloned into pET‐19 vector and overexpressed in *Escherichia coli BL21(DE3)*. The culture was incubated at 37 °C until the absorbance at 600 nm (OD600) reached 0.5–0.7. IPTG was added to a final concentration of 300 μM and overnight induction was performed at 18 °C. Cells were harvested by centrifugation. The bacterial pellet was resuspended in lysis buffer (50 mM NaH_2_PO_4_, pH 7.5, 300 mM NaCl, 0.1 mM PMSF) and lysed using a Microfluidizer (Microfluidics). A fresh portion of 0.1 mM PMSF and Triton X‐100 (1 % final concentration) were added. The lysate was cleared by ultracentrifugation at 30000 xg and 4 °C for 1 h. The protein purification was performed using immobilized nickel affinity chromatography (HisTrap, GE Healthcare) in a buffer containing 50 mM NaH_2_PO_4_ (pH 8), 300 mM NaCl and 5 % glycerol. Gradient elution was performed with a maximum of 0.5 M imidazole. Subsequently, the affinity tag was cleaved using His6‐TEV‐protease. The protease and unspecific binders were eliminated by a second nickel affinity chromatography. LIN28 A‐containing fractions were concentrated and applied to a High Load Superdex 75 pg 16/600 column (GE Healthcare) with a gel‐filtration buffer (30 mM NaH_2_PO_4_, pH 7.5, 50 mM NaCl, 5 % glycerol, 2 mM β‐ME). The purified protein was concentrated and stored at −80 °C.

**Fluorescence polarization (FP)**. The FP assay was conducted in black low‐volume polystyrene 384 well plates (Corning 4514). The inhibitory activity measurements were conducted in three technical replicates. The purified LIN28 A 16–187 was incubated at a concentration of 200 nM for 30 minutes with the compound (75 μM) in the FP assay buffer (20 mM Tris, pH 7, 100 mM NaCl, 5 mM MgCl_2_, 2 mM glutathione (reduced), 0.1 % NP‐40). Subsequently, FAM‐labeled preE*‐let‐7f–1* (mus musculus) (GGGGUAGUGAUUUUACCCUGUUUAGGAGAU‐FAM, synthesized by IDT) was added to a final concentration of 2 nM. Fluorescence polarization was detected after incubation at room temperature using a TECAN Spark plate reader.

**Chemistry**. All commercially purchased reagents and solvents were used without any further purification. The reaction was monitored by using thin‐layer chromatography (TLC) silica‐coated aluminum plates (Merck 60 F254) and visualization was achieved under UV irradiation (254 nm). Analytical UHPLC‐MS and LC–MS were performed on an Agilent 1260 II Infinity system (UHPLC column: Zorbax Eclipse C18 Rapid Resolution 2.1x50 mm 1.8 μm; LC–MS column: InfinityLab Poroshell 120 EC−C18, 2.1x150, 2.7 μm) and used the Acetonitrile (+ 0.1 % TFA) and Water (+ 0.1 % TFA) as the flow. Compounds were purified by either flash column chromatography (FC, silica gel 60 Å, 0.035–0.070 mm) or automated medium‐pressure liquid chromatography (MPLC, Buchi Pure C‐810, Buchi Pure C‐835). Alternatively, separations were carried out on a Buchi Pure C‐835 system (columns: Nuleodur C18 gravity VP 125/10 5 μm). Appropriate gradient systems obtained by mixing Acetonitrile (+ 0.1 % TFA) and Water (+ 0.1 % TFA) were used. NMR spectra were recorded on Bruker AV 400 Avance III HD (NanoBay), Agilent Technologies DD2, Bruker AV 500 Avance III HD (Prodigy), Bruker AV 600 Avance III HD (CryoProbe), or Bruker AV 700 Avance III HD (CryoProbe) spectrometers. Data was reported in parts per million (ppm) and used the deuterated solvent (CDCl_3_: 7.26 ppm, 77.16 ppm) as reference. Chemical shift value is reported in ppm, multiplicity (s=singlet, d=doublet, t=triplet, dd=double doublet, and m=multiplet), integration value, and coupling constant value in Hz. Mass spectrometry was performed on an LTQ Orbitrap mass spectrometer coupled to an Accela HPLC‐System (HPLC column: Hypersyl GOLD, 50 mm x 1 mm, particle size 1.9 μm, ionization method: electron spray ionization (ESI)). The synthetic procedures and the chemical and physical properties are included in the Supporting Information.

**Cell painting assay**. The cell painting assay was performed following the previously described methods.[^15^, ^16^] Initially, 5 μl U2OS medium was added to each well of a 384‐well plate (PerkinElmer CellCarrier‐384 Ultra). Subsequently, U2OS cells were seeded with a density of 1600 cells per well in 20 μL medium. The plate was incubated for 10 min at the ambient temperature, followed by an additional 4 h incubation (37 °C, 5 % CO2). Compound treatment was performed with the Echo 520 acoustic dispenser (Labcyte) at final concentrations of 10 μM, 3 μM or 1 μM. Incubation with the compound was performed for 20 h (37 °C, 5 % CO2). Subsequently, mitochondria were stained with Mito Tracker Deep Red (Thermo Fisher Scientific, Cat. No. M22426). The Mito Tracker Deep Red stock solution (1 mM) was diluted to a final concentration of 100 nM in a prewarmed medium. The medium was removed from the plate leaving 10 μl residual volume and 25 μl of the Mito Tracker solution was added to each well. The plate was incubated for 30 min in darkness (37 °C, 5 % CO_2_). To fix the cells 7 μl of 18.5 % formaldehyde in PBS was added, resulting in a final formaldehyde concentration of 3.7 %. Subsequently, the plate was incubated for another 20 min in darkness (RT) and washed three times with 70 μl of PBS. (Biotek Washer Elx405). Cells were permeabilized by the addition of 25 μl 0.1 % Triton X‐100 to each well, followed by 15 min incubation (RT) in darkness. The cells were washed three times with PBS leaving a final volume of 10 μl. To each well 25 μl of a staining solution was added, which contains 1 % BSA, 5 μl/ml Phalloidin (Alexa594 conjugate, Thermo Fisher Scientific, A12381), 25 μg/ml Concanavalin A (Alexa488 conjugate, Thermo Fisher Scientific, Cat. No. C11252), 5 μg/ml Hoechst 33342 (Sigma, Cat. No. B2261–25 mg), 1.5 μg/ml WGAAlexa594 conjugate (Thermo Fisher Scientific, Cat. No. W11262) and 1.5 μM SYTO 14 solution (Thermo Fisher Scientific, Cat. No. S7576). The plate is incubated for 30 min (RT) in darkness and washed three times with 70 μl PBS. After the final washing step, the PBS was not aspirated. The plates were sealed and centrifuged for 1 min at 500 rpm. The plates were prepared in triplicates with shifted layouts to reduce plate effects and imaged using a Micro XL High‐Content Screening System (Molecular Devices) in 5 channels (DAPI: Ex350–400/Em410–480; FITC: Ex470–500/Em510–540; Spectrum Gold: Ex520–545/Em560–585; TxRed: Ex535–585/Em600–650; Cy5: Ex605–650/Em670–715) with 9 sites per well and 20x magnification (binning 2). The generated images were processed with the *CellProfiler* package (https://cellprofiler.org/, version 3.0.0) on a computing cluster of the Max Planck Society to extract 1716 cell features per microscope site. The data was then further aggregated as medians per well (9 sites per 1 well), then over the three replicates. Further analysis was performed with custom *Python* (https://www.python.org/) scripts using the *Pandas* (https://pandas.pydata.org/) and *Dask* (https://dask.org/) data processing libraries as well as the *ScientificPython* (https://scipy.org/) package (separate publication to follow).

From the total set of 1716 features, a subset of highly reproducible and robust features was determined using the procedure described by Woehrmann et al.^[32]^ Two biological repeats of one plate containing reference compounds were analyzed. For every feature, its full profile over each whole plate was calculated. If the profiles from the two repeats showed a similarity of no less than 0.8 (see below), the feature was added to the set. This procedure was only performed once and resulted in a set of 579 robust features out of the total of 1716 that were used for all further analyses. The phenotypic profiles were compiled from the Z‐scores of all individual cellular features, where the Z‐score is a measure of how far away a data point is from a median value. Specifically, Z‐scores of test compounds were calculated relative to the Median of DMSO controls. The phenotypic compound profile is then determined as the list of Z‐scores of all features for one compound. In addition to the phenotypic profile, an induction value was determined for each compound as the fraction of significantly changed features, in percent. Similarities of phenotypic profiles (termed *Biosimilarity*) were calculated from the correlation distances (CD) between two profiles (https://docs.scipy.org/doc/scipy/ reference/generated/scipy.spatial.distance.correlation.html). The Biosimilarity is then defined as: *Biosimilarity*=1 – CD. Biosimilarity values smaller than 0 are set to 0 and the Biosimilarity is expressed in percent (0–100).

## Supporting Information Summary

No additional references are cited within the Supporting Information.

## Conflict of Interests

The authors declare no conflict of interest.

1

## Supporting information

As a service to our authors and readers, this journal provides supporting information supplied by the authors. Such materials are peer reviewed and may be re‐organized for online delivery, but are not copy‐edited or typeset. Technical support issues arising from supporting information (other than missing files) should be addressed to the authors.

Supporting Information

## Data Availability

The data that support the findings of this study are available from the corresponding author upon reasonable request.
