# Supplementary material for: LIN28‐Targeting Chromenopyrazoles and Tetrahydroquinolines Induced Cellular Morphological Changes and Showed High Biosimilarity with BRD PROTACs
Source: ChemMedChem. 2024 Nov 12;20(1):e202400547. doi: 10.1002/cmdc.202400547 (PMC11694606; doi:10.1002/cmdc.202400547)

ChemMedChem

Supporting Information

LIN28-Targeting Chromenopyrazoles and Tetrahydroquinolines Induced Cellular Morphological Changes and Showed High Biosimilarity with BRD PROTACs

Mao Jiang, Nicole Giannino, Georg L. Goebel, Sonja Sievers, and Peng Wu*

SUPPORTING INFORMATION

LIN28-Targeting Chromenopyrazoles and Tetrahydroquinolines Induced Cellular Morphological Changes and Showed High Biosimilarity with BRD PROTACs

Mao Jiang,[a,b,c] Nicole Giannino,[a,b] Georg L. Goebel,[a,b,c] Sonja Sievers,[b,d] and Peng Wu*[a,b,c]

[a] Chemical Genomics Centre, Max Planck Institute of Molecular Physiology, Otto-Hahn Str. 11, Dortmund 44227, Germany

[b] Department of Chemical Biology, Max Planck Institute of Molecular Physiology, Otto-Hahn Str. 11, Dortmund 44227, Germany

[c] Faculty of Chemistry and Chemical Biology, TU Dortmund University, Otto-Hahn Str. 6, Dortmund 44227, Germany

[d] Compound Management and Screening Center, Otto-Hahn Str. 15, Dortmund 44227, Germany

*Correspondence, P. Wu, email: peng.wu@mpi-dortmund.mpg.de

SUPPLEMENTARY FIGURES

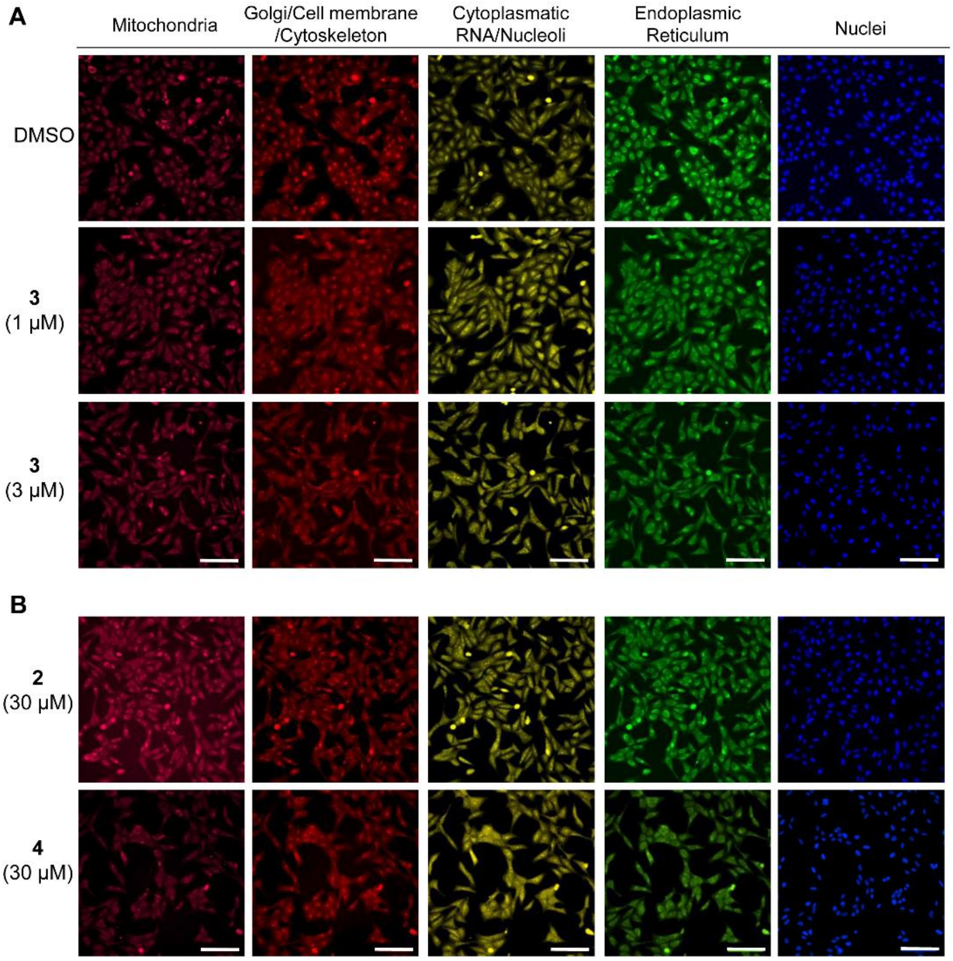

Figure S1. Selected images from the CPA analysis showing the cellular morphological change of U2OS cells upon treatment with the CMPs. (A) Treatment with compound **3** at 1 and 3 μM. (B) Treatment with compounds **2** and **4** at 30 μM. DMSO was used as the control. Scale bar, 150 μm for all images.

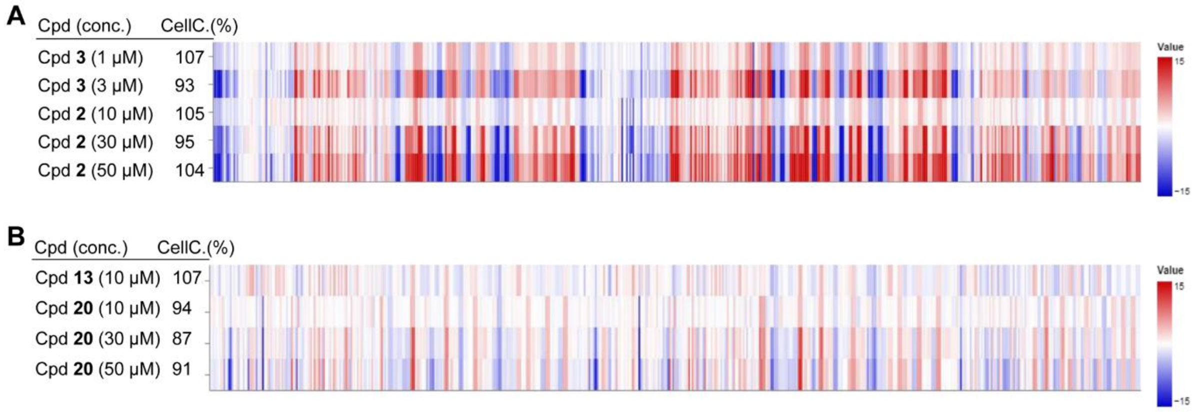

Figure S2. Heapmap showing the biosimilarity fingerprints in the CPA analysis for the selected CMPs (A) and THQs (B). The relative cell count (CellC.%) values indicated that the tested compounds showed no cytoxicity at the testing concentrations ranging from 1 µM to 50 µM. Values were normalized to that of the DMSO control. Blue: decreased parameters; red: increased parameters.

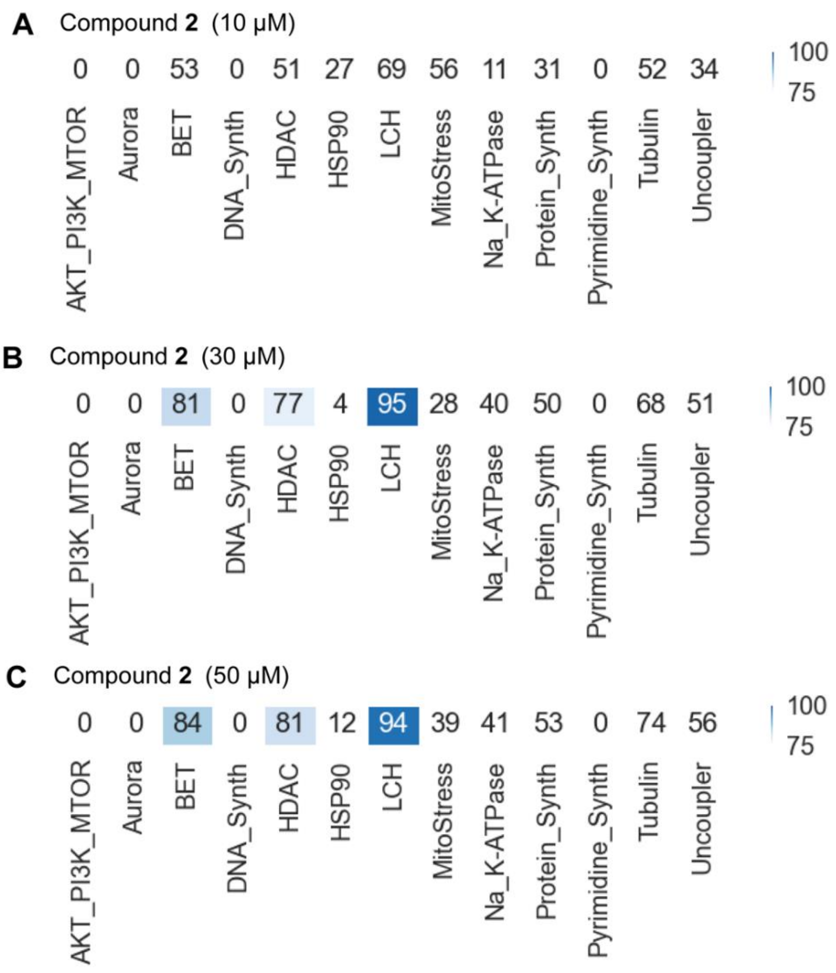

Figure S3. Subprofile analysis of the CMP compound **2** with established bioactivity clusters. Compound **2** showed a high biosimilarity with the LCH and BET clusters at both 30 and 50 µM, while no significant biosimilarity was observed with any of the established clusters at 10 µM.

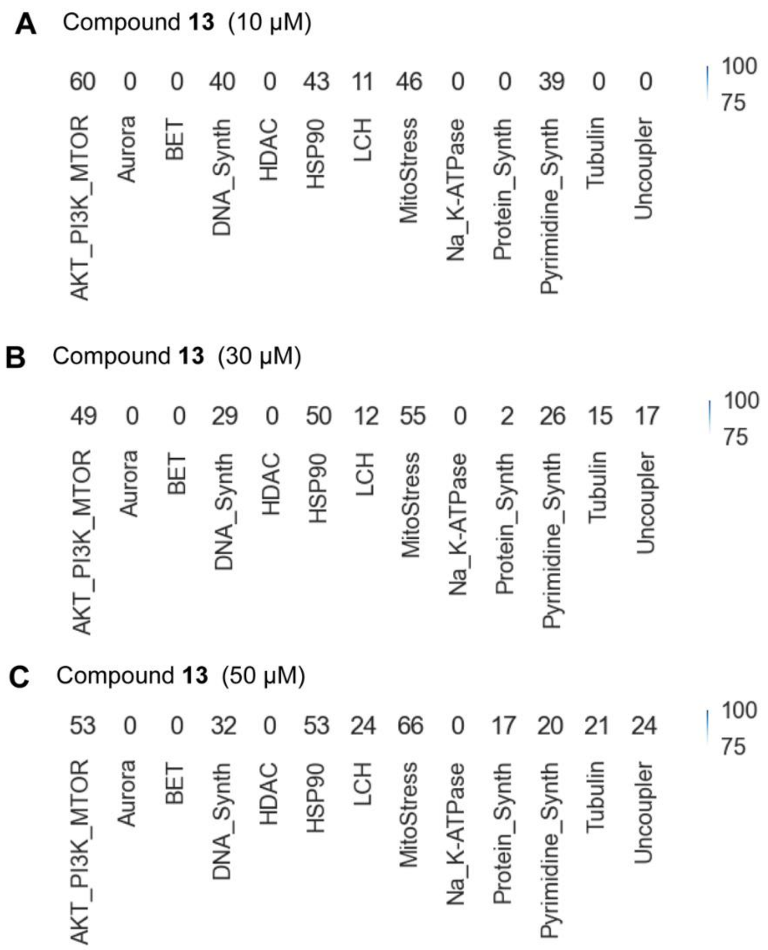

Figure S4. Subprofile analysis of the THQ compound **13** with established bioactivity clusters. Compound **13** did not show a high biosimilarity (>85%) with any of the established bioactivity clusters at the three tested concentrations ranging from 10 to 50 µM.

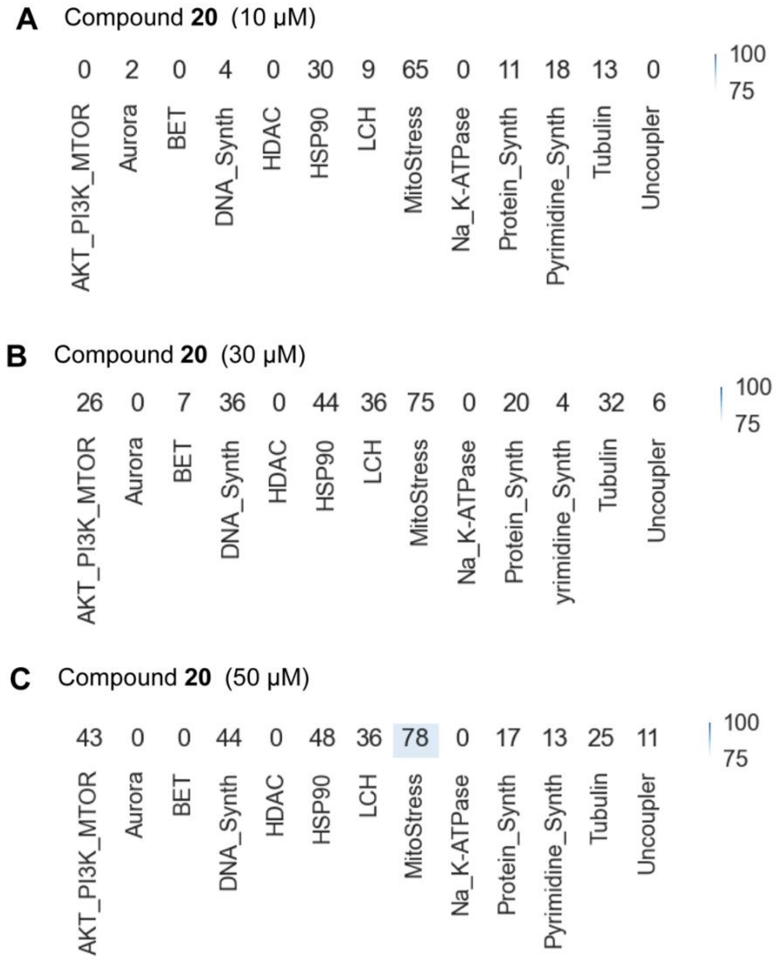

Figure S5. Subprofile analysis of the THQ compound **20** with established bioactivity clusters. Compound **20** did not show a high biosimilarity (>85%) with any of the established bioactivity clusters at the three tested concentrations ranging from 10 to 50 µM.

SYNTHETIC PROCEDURES AND COMPOUND CHARACTERIZATION

1-(4-Fluoro-2-hydroxy-5-nitrophenyl)ethan-1-one (I-1)

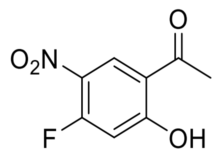

A mixture of 70% HNO_3 (1.75 g, 1.24 ml, 19.46 mmol, 1.5 eq.) and conc. H_2SO_4 was added dropwise into a mixture of hydroxy acetophenone (2.00 g, 12.98 mmol, 1 eq.) in 6 mL conc. H_2SO_4 at -5°C. The mixture was stirred for an additional 30 min at 0 °C. The reaction was monitored by TLC. Upon completion of the reaction, the mixture was poured into ice water and the crude residue was filtered and washed. The crude was purified through silica gel chromatography to obtain the desired product **I-1** (1.12 g, 43%). ^{1}H NMR (400MHz, Chloroform-*d*) δ 13.01 (d, J = 1.4 Hz, 1H), 8.65 (d, J = 8.3 Hz, 1H), 6.83 (d, J = 12.1 Hz, 1H), 2.71 (3H). LC-MS (ESI) (*m/z*): $[M+H]^+$ calculated for $C_8H_7FNO_4$: 200.0; found: 200.2.

Benzyl 4-(4-acetyl-5-hydroxy-2-nitrophenyl)piperazine-1-carboxylate (I-2)

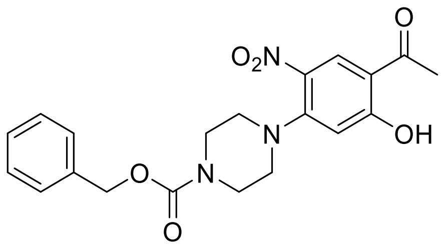

Compound **I-1** (1.00 g, 5.02 mmol, 1.0 eq.) was dissolved in 30 mL acetonitrile, followed by the addition of Cbz-piperazine (1.33 g, 6.25 mmol, 1.2 eq.) in one portion. The mixture was stirred overnight at 40 °C. After the completion of the reaction as monitored by TLC, the solvent was removed under reduced pressure. The crude was dissolved in DCM. The organic phase was washed with 1 N HCl (aq), brine and dried

over anhydrous $MgSO_4$. The filtrate was dried under reduced pressure to yield the desired product **I-2** without further purification (1.98 g, 99%): ^{1}H NMR (700 MHz, Chloroform-*d*) δ 12.82 (s, 1H), 8.44 (s, 1H), 7.40 – 7.36 (m, 4H), 7.33 (ddd, J = 8.5, 3.9, 2.3 Hz, 1H), 6.43 (s, 1H), 5.17 (s, 2H), 3.73 – 3.67 (m, 4H), 3.16 (s, 4H), 2.60 (s, 3H). ^{13}C NMR (176 MHz, Chloroform-*d*) δ 202.39, 166.77, 155.30, 152.25, 136.52, 132.86, 132.24, 128.73 (2C), 128.37, 128.18 (2C), 112.63, 106.50, 67.65, 50.72 (2C), 43.37 (2C), 26.29. LC-MS (ESI) (*m/z*): $[M+H]^+$ calculated for $C_{20}H_{22}N_3O_6$: 400.2; found: 400.2.

Benzyl 4-(1'-methyl-6-nitro-4-oxospiro[chromane-2,4'-piperidin]-7-yl)piperazine-1-carboxylate (I-3)

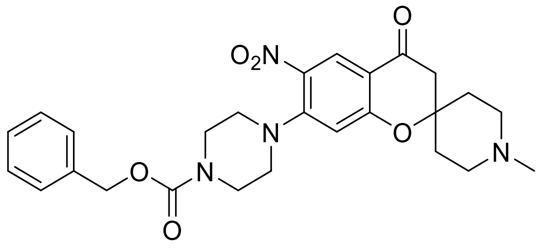

Compound **I-2** (1.50 g, 3.76 mmol, 1.0 eq.) dissolved in 10 mL DMSO was

A mixture of pyrrolidine (0.56 g, 7.89 mmol, 2.1eq.) and butyric acid (0.69 g, 7.89 mmol, 2.1 eq.) were added in one portion to 1-methylpiperidin-4-one (0.47 g, 4.13 mmol, 1.1 eq.) dissolved in 10 mL DMSO. The resulting reaction mixture was stirred at room temperature for 15 min, followed by the addition of compound **I-2** (1.50 g, 3.76 mmol, 1.0 eq.) dissolved in 10 mL DMSO. Upon completion of the reaction monitored by TLC, the reaction mixture was poured into water. The aqueous phase was extracted by ethyl acetate (30 mL) three times. The combined organic phase was washed with brine and dried over anhydrous $MgSO_4$, filtered, and concentrated under reduced

pressure. The resultant residue was purified by silica gel flash column chromatography to yield the desired product **I-3** (0.97 g, 52%): ^{1}H NMR (700 MHz, Chloroform-*d*) δ 8.46 (s, 1H), 7.39 – 7.36 (m, 4H), 7.35 – 7.32 (m, 1H), 6.48 (s, 1H), 5.17 (s, 2H), 3.74 – 3.66 (m, 4H), 3.15 (d, *J* = 35.6 Hz, 4H), 2.70 (s, 2H), 2.64 (d, *J* = 9.8 Hz, 2H), 2.44 (t, *J* = 10.8 Hz, 2H), 2.35 (s, 3H), 2.05 (s, 2H), 1.86 – 1.79 (m, 2H); LC-MS (ESI) (*m/z*): $[M+H]^+$ calculated for $C_{26}H_{31}N_4O_6$: 495.2; found: 495.5.

Benzyl 4-(3-(diethoxymethyl)-1'-methyl-6-nitro-4-oxospiro[chromane-2,4'-piperidin]-7-yl)piperazine-1-carboxylate (I-4)

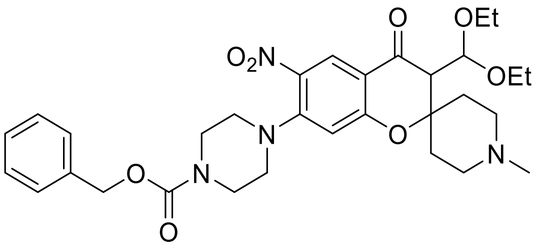

To a solution of triethyl orthoformate (0.53 g, 3.64 mmol, 3.0 eq.) in dry DCM, $BF_3{\cdot}OEt_2$ (4.5 mL, 3.64 mmol, 3.0 eq.) was slowly added over a period of 10 min at -10 °C under an argon atmosphere. The reaction mixture was then gradually warmed up to 0 °C. After 15 min of stirring at 0 °C, the reaction mixture was cooled back to -78 °C. To this reaction mixture, Compound **I-3** (0.60 g, 1.21 mmol, 1.0 eq.) in DCM was added, and DIPEA (0.55 g, 4.25 mmol, 3.5 eq.) was then added slowly over 30 min. The resulting mixture was stirred at -78 °C for 10 min, and then warmed up to room temperature for an additional 2 hours of stirring. The resultant mixture was diluted with DCM and washed with sat. $NaHCO_3$(aq) solution. The combined organic layer was dried over anhydrous $MgSO_4$ and condensed under reduced pressure. The resulting mixture was purified by silica gel flash column chromatography to provide the desired product **I-4**

(0.49 g, 68%). LC-MS (ESI) (*m/z*): $[M+H]^+$ calculated for $C_{31}H_{41}N_4O_8$: 597.3; found: 597.6.

Benzyl (Z)-4-(3-(hydroxymethylene)-1'-methyl-6-nitro-4-oxospiro[chromane-2,4'-piperidin]-7-yl)piperazine-1-carboxylate (I-5)

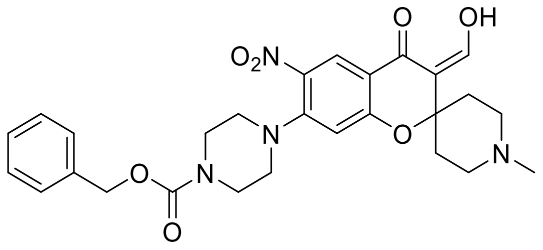

Iodine (18.7 mg, 0.07 mmol, 0.2 eq.) was added in one portion to a solution of compound **I-4** (0.22 g, 0.37 mmol, 1.0 eq.) in acetone. The resulting reaction mixture was stirred at 35 °C for 16 hours. Upon completion of the reaction as monitored by TLC, the solvent was removed under reduced pressure. The resulting crude was dissolved in DCM and washed sequentially with 5% aqueous $Na_2S_2O_3$, H_2O, and brine. The organic phase was dried over anhydrous $MgSO_4$ and filtered. The filtrate was condensed under reduced pressure, and the resulting residue was purified by silica gel flash column chromatography to yield the desired product **I-5** (123.7 mg, 86%). LC-MS (ESI) (*m/z*): $[M+H]^+$ calculated for $C_{27}H_{31}N_4O_7$: 523.2; found: 523.7.

4-(7-(4-((benzyloxy)carbonyl)piperazin-1-yl)-1'-methyl-8-nitro-1H-spiro [chromeno[4,3-c]pyrazole-4,4'-piperidin]-1-yl)benzoic acid (2)

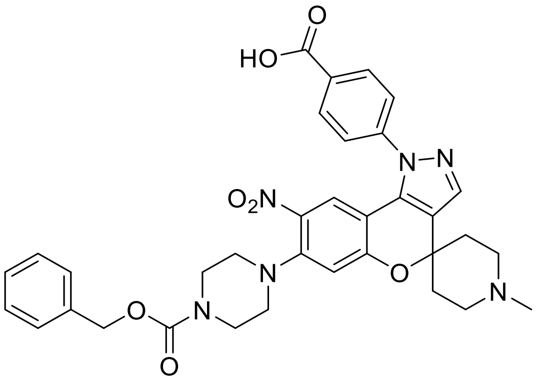

To a solution of 4-hydrazineylbenzoic acid (17.5 mg, 0.12 mmol, 1.2 eq.) in AcOH, compound **I-5** (50.0 mg, 0.10 mmol, 1.0 eq.) was added carefully and the mixture was stirred for 2 hours at 35 °C. The solvent was removed under vacuum conditions. The residue was purified by silica gel flash column chromatography to yield the desired product **2** (12.2 mg, 20%). ^{1}H NMR (500 MHz, Chloroform-*d*) δ 13.41 (s, 1H), 8.26 (d, *J* = 8.5 Hz, 2H), 7.66 (s, 1H), 7.63 – 7.58 (m, 3H), 7.38 – 7.32 (m, 5H), 6.67 (s, 1H), 5.16 (s, 2H), 3.69 (t, *J* = 4.4 Hz, 4H), 3.61 (d, *J* = 10.1 Hz, 2H), 3.26 (t, *J* = 12.5 Hz, 2H), 3.08 (br, 4H), 2.95 (s, 3H), 2.66 (td, *J* = 14.5, 4.1 Hz, 2H), 2.33 (d, *J* = 14.5 Hz, 2H). ^{13}C NMR (126 MHz, $CDCl_3$) δ 168.25, 163.07, 155.66, 155.40, 151.38, 148.45, 143.38, 136.54, 136.07, 135.48, 131.84, 130.86, 130.49, 128.75, 128.40, 128.17, 125.66, 122.11, 119.81, 108.98, 108.36, 75.13, 67.65, 50.08, 43.94, 33.90. LC-MS (ESI) (*m/z*): $[M+H]^+$ calculated for $C_{34}H_{35}N_6O_7$: 639.3; found: 639.6.

Benzyl 4-(1'-methyl-8-nitro-1-phenyl-1H-spiro[chromeno[4,3-c]pyrazole-4,4'-piperidin]-7-yl)piperazine-1-carboxylate (3)

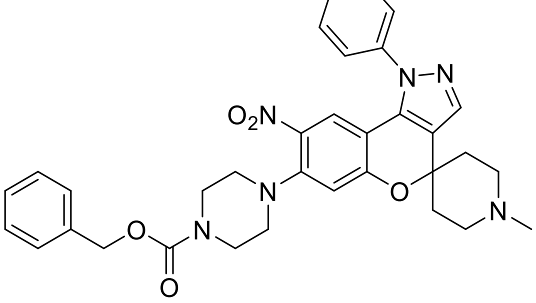

Compound **I-5** (50.0 mg, 0.10 mmol, 1.0 eq.) was added carefully to a solution of phenyl hydrazine (12.4 mg, 0.12 mmol, 1.2 eq.) in AcOH. The resulting mixture was stirred for 2 hours at 35 °C. The solvent was removed under vacuum conditions. The residue was purified by silica gel flash column chromatography to yield the desired product **3** (27.3 mg, 48%). ^{1}H NMR (700 MHz, Chloroform-*d*) δ 7.60 (s, 1H), 7.59 – 7.56 (m, 3H), 7.48 (s, 1H), 7.48 – 7.45 (m, 2H), 7.38 – 7.33 (m, 5H), 6.64 (s, 1H), 5.16 (s, 2H), 3.72 – 3.66 (m, 4H), 3.66 – 3.60 (m, 2H), 3.29 (t, *J* = 12.1 Hz, 2H), 3.13 – 2.99 (m, 4H), 2.96 (s, 3H), 2.60 (td, *J* = 14.9, 4.3 Hz, 2H), 2.36 – 2.35 (m, 2H). ^{13}C NMR (176 MHz, $CDCl_3$) δ 161.22, 161.00, 155.40, 155.30, 148.15, 139.04, 136.34, 136.11, 134.26, 130.71, 130.02, 129.88, 128.60, 128.27, 128.03, 126.06, 121.88, 118.32, 108.66, 108.44, 74.84, 67.54, 50.16, 43.89, 33.98. LC-MS (ESI) (*m/z*): $[M+H]^+$ calculated for $C_{33}H_{35}N_6O_5$: 595.3; found: 595.6.

NMR SPECTRA

^{1}H NMR spectrum of compound 2 (500 MHz, Chloroform-*d*):

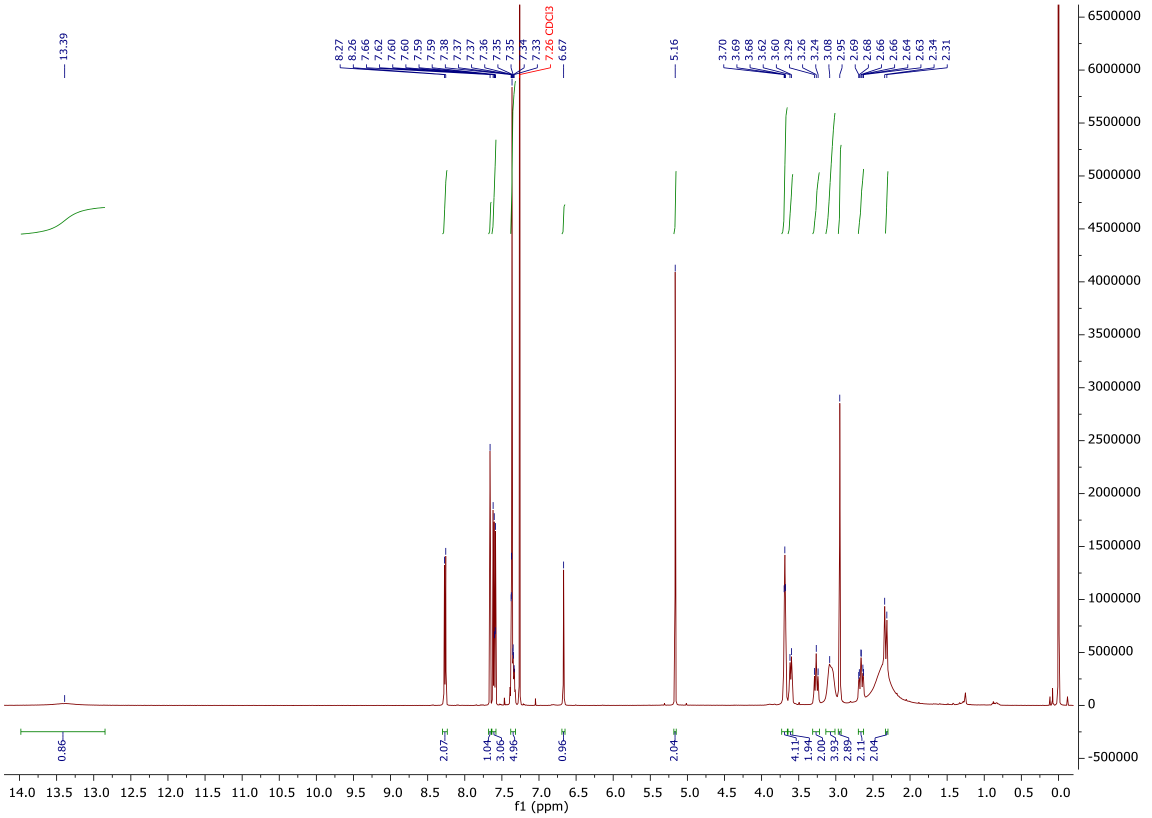

^{13}C NMR spectrum of compound 2 (126 MHz, Chloroform-*d*):

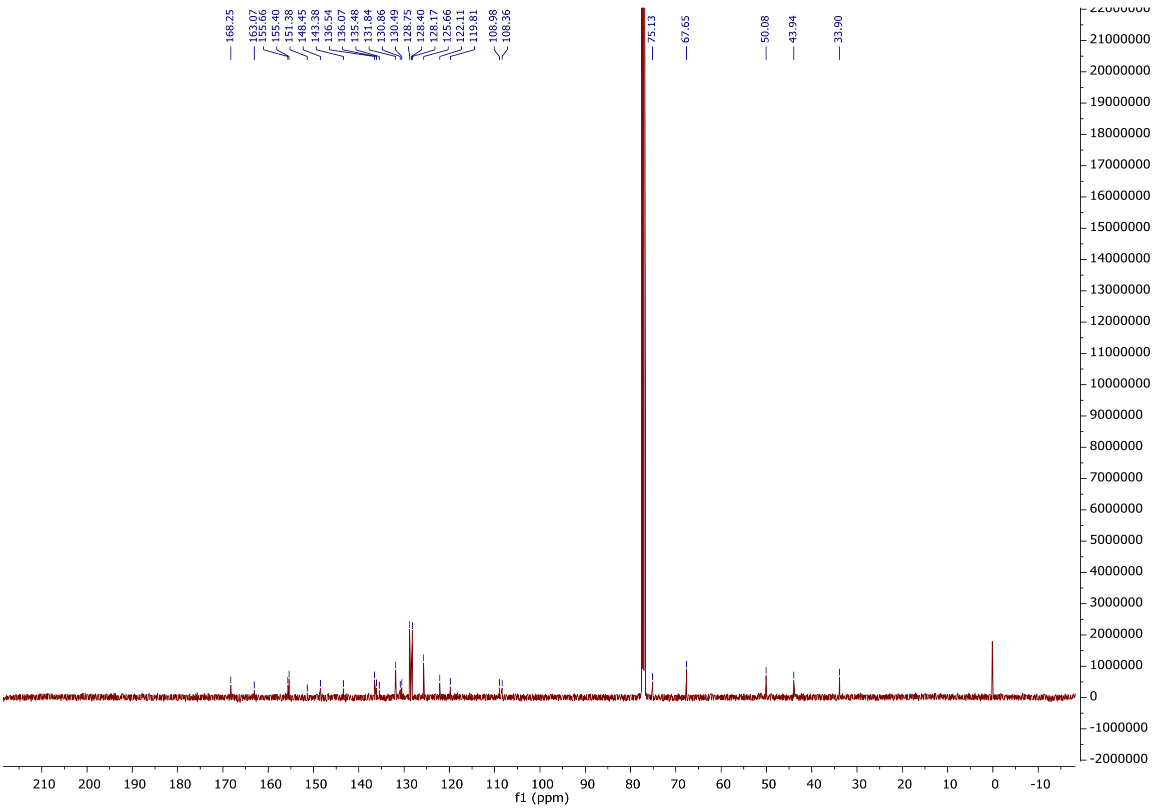

^{1}H NMR spectrum of compound 3 (700 MHz, Chloroform-*d*):

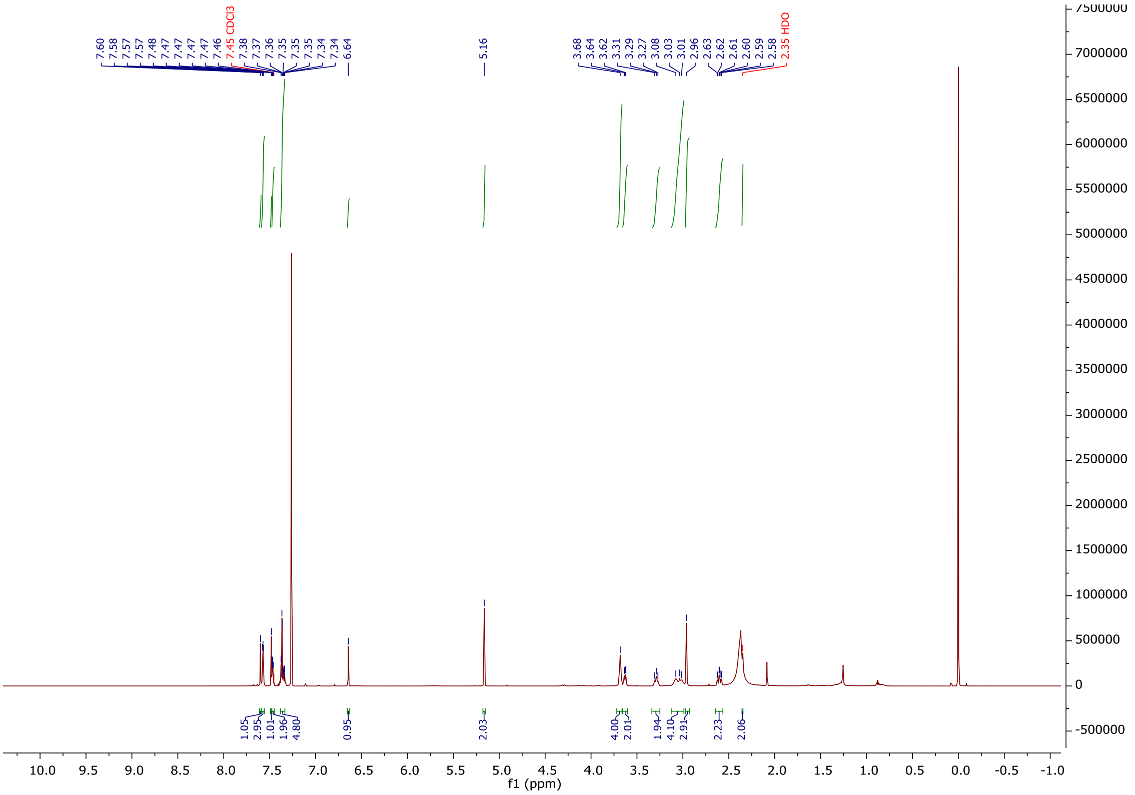

^{13}C NMR spectrum of compound 3 (176 MHz, Chloroform-*d*):

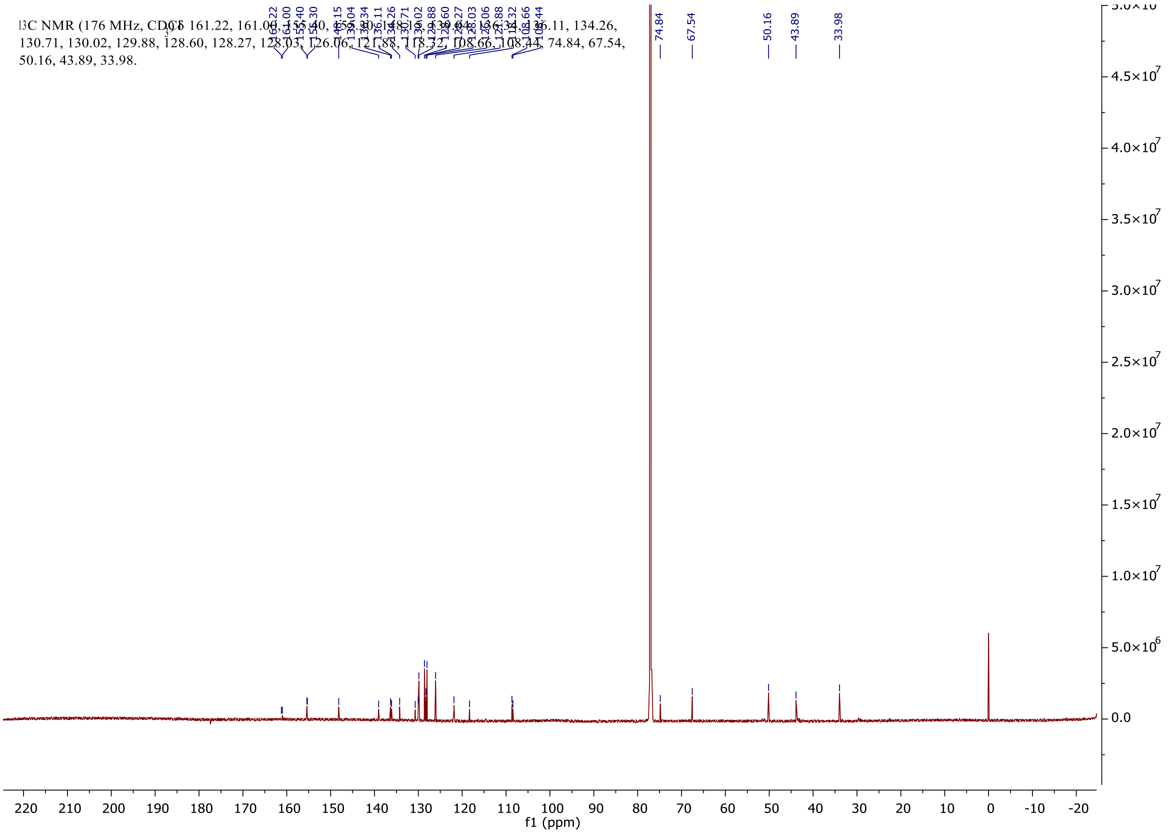

Supplement: Supplementary file 1 — Supporting Information [file CMDC-20-e202400547-s001.pdf]
